# Single Session Low Frequency Left Dorsolateral Prefrontal Transcranial Magnetic Stimulation Changes Neurometabolite Relationships in Healthy Humans

**DOI:** 10.3389/fnhum.2018.00077

**Published:** 2018-03-26

**Authors:** Nathaniel R. Bridges, Richard A. McKinley, Danielle Boeke, Matthew S. Sherwood, Jason G. Parker, Lindsey K. McIntire, Justin M. Nelson, Catherine Fletchall, Natasha Alexander, Amanda McConnell, Chuck Goodyear, Jeremy T. Nelson

**Affiliations:** ^1^Infoscitex Inc., Dayton, OH, United States; ^2^Warfighter Interfaces Division, Applied Neuroscience Branch, Wright-Patterson AFB (WPAFB), Dayton, OH, United States; ^3^Wright State Research Institute, Wright State University, Dayton, OH, United States; ^4^Kettering Health Network Innovation Center, Kettering, OH, United States; ^5^Grandview Medical Center, Kettering Health Network, Dayton, OH, United States; ^6^Research Imaging Institute, School of Medicine, University of Texas Health Science Center, San Antonio, San Antonio, TX, United States

**Keywords:** transcranial magnetic stimulation (TMS), dorsolateral prefrontal cortex (DLPFC), low frequency, magnetic resonance spectroscopy, sternberg task, healthy subjects

## Abstract

**Background**: Dorsolateral prefrontal cortex (DLPFC) low frequency repetitive transcranial magnetic stimulation (LF-rTMS) has shown promise as a treatment and investigative tool in the medical and research communities. Researchers have made significant progress elucidating DLPFC LF-rTMS effects—primarily in individuals with psychiatric disorders. However, more efforts investigating underlying molecular changes and establishing links to functional and behavioral outcomes in healthy humans are needed.

**Objective**: We aimed to quantify neuromolecular changes and relate these to functional changes following a single session of DLPFC LF-rTMS in healthy participants.

**Methods**: Eleven participants received sham-controlled neuronavigated 1 Hz rTMS to the region most activated by a 7-letter Sternberg working memory task (SWMT) within the left DLPFC. We quantified SWMT performance, functional magnetic resonance activation and proton Magnetic resonance spectroscopy (MRS) neurometabolite measure changes before and after stimulation.

**Results**: A single LF-rTMS session was not sufficient to change DLPFC neurometabolite levels and these changes did not correlate with DLPFC activation changes. Real rTMS, however, significantly altered neurometabolite correlations (compared to sham rTMS), both with baseline levels and between the metabolites themselves. Additionally, real rTMS was associated with diminished reaction time (RT) performance improvements and increased activation within the motor, somatosensory and lateral occipital cortices.

**Conclusion**: These results show that a single session of LF-rTMS is sufficient to influence metabolite relationships and causes widespread activation in healthy humans. Investigating correlational relationships may provide insight into mechanisms underlying LF-rTMS.

## Introduction

Within the past decade, low frequency repetitive transcranial magnetic stimulation (LF-rTMS, typically delivered at 1 Hz) applied to the dorsolateral prefrontal cortex (DLPFC; Dayan et al., 2013) has shown potential in treating a broad spectrum of clinical diseases/disorders. Some examples include depression (Brunelin et al., 2014), autism (Casanova et al., 2014), pain (Sampson et al., 2011), post-traumatic stress disorder (Berlim and Van den Eynde, 2014), and Parkinson’s disease (Nardone et al., 2014). Additionally, it has been used as a tool to gain insight into brain function (e.g., Balconi, 2013; Dienes and Hutton, 2013) and has demonstrated potential to enhance cognition in healthy human populations (Luber and Lisanby, 2014).

It is well known that LF-rTMS decreases cortical excitability (Chen et al., 1997), which researchers have primarily assessed in the motor cortex (M1). M1 LF-rTMS has accordingly resulted in activation decreases at the stimulation site, measured using functional magnetic resonance imaging (fMRI; Nowak et al., 2008). Similarly, DLPFC LF-TMS has resulted in decreased oxygenated hemoglobin levels at the stimulation site (Kozel et al., 2009) as well as altered fMRI activation (van der Werf et al., 2010) and cortical potentials (De Ridder et al., 2013) in remote brain regions. These results generally suggest that DLPFC rTMS causes decreased activation at the stimulation site, but that these changes can influence functionally connected remote brain regions.

The ability of TMS to selectively target the DLPFC, a region thought to exert top-down modulation of networks engaged in working memory (Zanto et al., 2011), has the potential to serve as a valuable tool in modulating working memory processes. However, the behavioral impact of DLPFC LF-rTMS on working memory processes is not clear (Lage et al., 2016). For example, DLPFC LF-rTMS interfered with working memory in one study (Škrdlantová et al., 2005), enhanced it in others (Fregni et al., 2006a; Fitzgerald et al., 2009) and had no effect in another set of experiments (Hoffman et al., 2005; Kang et al., 2009; Kim et al., 2010; Watts et al., 2012). Differences in these results are likely due to the heterogeneity across the populations investigated as they were studied in groups with various mental disorders/psychiatric illnesses. Studies investigating mechanisms underlying these changes in healthy human populations would therefore be helpful in explaining the varying behavioral outcomes across non-healthy populations.

Magnetic resonance spectroscopy (MRS), which can be used to measure and detect changes in neurometabolites and relate these changes to behavioral and activation measures, is a valuable tool for exploring such mechanisms. In particular, glutamate/glutamine (Glx), an excitatory neurotransmitter/neurotransmitter precursor, whose levels can be quantified using MRS, has been positively correlated with various rTMS-measures of cortical excitability (Stagg et al., 2011; Tremblay et al., 2013; Lewis et al., 2016) and working memory performance (Marsman et al., 2017; Vijayakumari et al., 2018). Much like Glx, N-acetylaspartate (NAA), which can also be measured with MRS, has also been positively related to working memory performance (Bertolino et al., 2000, 2003; Ozturk et al., 2009; Erickson et al., 2012; Mao et al., 2016).

To the authors’ knowledge, however, no studies have used MRS to quantify metabolites in the DLPFC following LF-rTMS. Instead, research has focused on high frequency (HF)-rTMS—typically 5 Hz or higher (e.g., Brighina et al., 2004; Conforto et al., 2013), applied to this region. HF-rTMS DLPFC rTMS, for example, has resulted in stimulation site increases in Glx (Michael et al., 2003; Luborzewski et al., 2007; Yang et al., 2014; Aleman and Dlabac-de Lange, 2016; Dlabac-de Lange et al., 2017) as well as NAA levels (Aleman and Dlabac-de Lange, 2016). As such, our primary aim was to quantify neuromolecular changes following a single session of DLPFC LF-rTMS in healthy participants. Given the relationships Glx and NAA have with working memory performance as well as rTMS-effects, we elected to primarily focus on Glx and NAA neuromolecular changes. To quantify these neuromolecules/neurometabolites, we used proton MRS (^1^H MRS) measured at 1.5 T. These measures were coupled with cortical activity produced during a working memory task. While known that it is difficult to completely parse Glx from the major inhibitory neurotransmitter γ-Aminobutyric acid (GABA) using this magnetic field strength, GABA is generally much lower than glutamate levels which dominates the Glx signal (see Michael et al., 2003). As such, given that excitatory HF-rTMS results in increased Glx and NAA levels and that Glx and NAA levels positively correlate with working memory performance in the DLPFC, we hypothesized that LF-rTMS—which is generally inhibitory, would result in decreased in Glx and NAA levels and working memory performance. Further, given that a single session of HF DLPFC rTMS was sufficient to change neuormetabolite levels in healthy humans (Michael et al., 2003), we hypothesized that a single session of LF DLPFC rTMS would also be sufficient to cause Glx and NAA changes in healthy humans. Finally, it is well known that increased excitatory neurotransmitter levels can increase firing rate activity. Therefore, we expected that MRS-assessed neurometabolite levels would positively correlate with BOLD-fMRI, which has been recently shown (Betina Ip et al., 2017). If rTMS decreased Glx and NAA levels, as hypothesized, we would therefore expect changes in these metabolites to correlate with BOLD-fMRI activation levels.

## Materials and Methods

### Participants

Eleven healthy active duty military members participated in this study (10 males and 1 female; mean age of 29.6 ± 6.2 years). Two subjects were left handed while the remaining were right handed. All were recruited through Wright-Patterson Air Force Base and enrolled after obtaining written informed consent in accordance with the Declaration of Helsinki. Two local ethics committees approved this study’s protocol including the 711th Human Performance Wing and Copernicus Institutional Review Board. Exclusion criteria included: participants outside the ages of 18 and 42, known or first degree relatives presenting neurological disorders or abnormalities, head injuries or concussions, a history of alcohol/drug abuse, diagnosed forms of heart disease or high-blood pressure, sleep disorders, pregnancy and participants consuming medications or supplements known to lower the seizure threshold (see Rossi et al., 2009). Participants were asked to receive at least 6 h of sleep and avoid alcohol consumption within 24 h of receiving rTMS. Study participants were additionally required to pass a neurological evaluation before and after stimulation.

### Procedure

**Figure 1:**
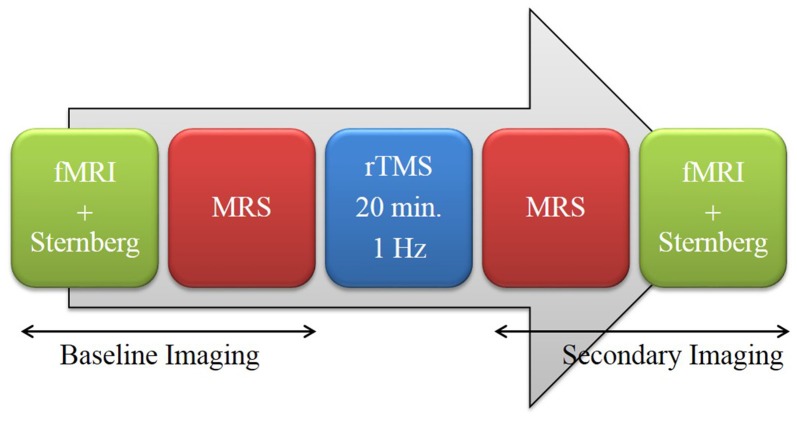
Procedural Outline.

Following informed consent and screening, participants received baseline magnetic resonance imaging (MRI). On a typical experimental day, participants underwent imaging before and after rTMS. “Baseline Imaging”, which occurred prior to rTMS, consisted of fMRI with a concurrent 7-letter Sternberg working memory task (SWMT) followed by single voxel ^1^H MRS. “Secondary Imaging” used the same imaging modalities but in reverse order—MRS followed by fMRI with the task. The order of pre and post-TMS imaging modalities were designed such that MRS occurred immediately before and after TMS (Figure 1). Participants experienced either real or sham rTMS counterbalanced across 2 days (resulting in “sham-real” or “real-sham” groups). To minimize carry-over effects, a minimum of 7 days was required before the second experimental session took place. The median number of days between experimental sessions was 27 with a minimum and maximum of 7 and 97 days respectively.

### rTMS

Twenty minutes of continuous 1 Hz neuronavigated rTMS was delivered to the most functionally active region of the left DLPFC identified during baseline imaging (on the first experimental day). Neuronavigation increases cortical accuracy and optimal coil placement resulting in improved functional outcomes (Ruohonen and Karhu, 2010; Takahashi et al., 2013; Kim et al., 2014). The MagPro R30 (MagVenture, Denmark) MCF-B65 butterfly head (sham = MCF-P-B65) and Brainsight system (Rogue Research Inc., Montreal, QC Canada) were used for rTMS and neuronavigation respectively. The sham and real TMS coils were identical in appearance, auditory output and placement location. Sham coils, however did not induce current at the target site. The handle was oriented such that it was perpendicular to the longitudinal axis of the target gyrus within the region of activation. Stimulation intensity was provided at 100% resting motor threshold (rMT), defined as the lowest stimulation intensity at which 5 out of 10 TMS pulses produced visual responses in the abductor pollicis brevis muscle of the contralateral hand (Greenberg et al., 1997). rMT was determined by stimulating the hemisphere contralateral to the subject’s dominant hand.

### Experimental Task

Delivery of the SWMT within the MR scanner was accomplished using a MR-compatible visual projection system (BrainLogics MRI Digital Projection System; Psychology Software Tools Inc., Sharpsburg, PA, USA) coupled to a mirror located at the top of a standard 12-channel bird-cage head coil. Participants were required to lay in a supine position, with their hands to the side and their head held in place with the bird-cage head coil. During the task, participants were asked to judge whether a test letter was contained in a previously memorized sequence of letters. On the first experimental day participants were given a short practice session to familiarize them to the task before beginning. Reaction times (RTs) and response types were recorded for each stimulus.

The SWMT was executed in a boxcar design alternating 16 s control blocks with 16 s task blocks (adapted from Caldwell et al., 2005). During the task blocks, participants were required to provide either a “yes” or “no” answer if an alphabetic letter was or was not contained in a 7-letter recognition set of randomized alphabetic consonants. The block began by displaying a 7-letter string for 3000 ms, referred to as the encoding phase. After a 7000 ms delay, referred to as the retention period, a test letter (*i.e*., probe) appeared for 3000 ms. The participant was given 6000 ms to respond to the probe before the start of the next block. This response window began at the onset of the probe, resulting in a total block duration of 16 s. The control block was identical except participants’ “yes” or “no” answer was in response to “Y” or “N” probes to non-alphanumeric character recognition set. Participants completed 12 task and control blocks for a total duration of 6 min 24 s.

### MRI Acquisition

All participants underwent identical MR procedures before and after brain stimulation using a Siemens MAGNETOM Avanto 1.5T MRI scanner (Siemens Medical Systems, Erlangen, Germany). The imaging sequences began with a high-resolution T1-weighted anatomical scan acquired using a 3D magnetization-prepared rapid acquisition gradient-echo (MPRAGE) sequence with a 512 × 512 element matrix, 120 slices, 1 × 1 × 1 mm voxel size, TR/TE = 500/15 ms, and flip angle = 15°. A single fMRI acquisition was then acquired using a gradient recalled echo sequence with a 64 × 64 element matrix, 24 slices, 4.5 × 4.5 × 5 mm voxel size, 1 mm slice gap, TR/TE = 2000/10 ms, and flip angle = 90°. The SWMT was synchronized to the pulse sequence using a 5-V transistor-transistor logic pulse received from the MRI at the start of every new TR. A total of 192 volumes were acquired for an acquisition time of 6 min 24 s. Single-voxel ^1^H MRS (SVS) measurements were then performed using a point resolved spectroscopy (PRESS) pulse sequence with TR = 1500 ms, TE = 135 ms, # of averages = 4, flip angle = 90°, and voxel dimensions = 20.0 × 20.0 × 20.0 mm^3^. Transmitter and receiver gains and the center frequency were automatically adjusted during pre-scanning. A three-plane auto-shim procedure optimized the local magnetic field homogeneity, and the flip angle of the third water suppression pulse was adjusted for chemical-shift-water suppression (CHESS) prior to the acquisition. The SVS voxel was centered in an activation cluster of the left DLPFC localized by the fMRI parameter estimate maps.

### MRS

Four neurometabolites were measured for each session: glutamate/glutamine (Glx), choline (Cho), which is associated with increases in membrane turnover, creatine (Cr), associated with energy usage, and total N-acetylaspartate (tNAA), which is comprised of NAA, a marker of neuronal viability and N-acetylaspartateglutamate (NAAG), a neurotransmitter derivative of NAA and glutamate (Ross and Sachdev, 2004; Ende, 2015). Glx, Cho and tNAA metabolite values were normalized to Cr to create Glx/Cr, Cho/Cr and tNAA/Cr ratios for additional analysis. Since the primary objective of this study was to assess neurometabolite changes following LF-rTMS and research suggested that rTMS after-effects last approximately the duration of stimulation (Rossi et al., 2009), we put great effort into ensuring that the time from the start of rTMS to the end of MRS acquisition was approximately 20 min or less. This duration was 20 min or less for 19 of the total 22 imaging sessions (11 post real and 11 post-sham). The remaining three sessions ranged from 21 min to 24 min. While absolute metabolite quantification methods were considered, we elected to use ratios to minimize the post-rTMS MRS acquisition time in an effort to capture expected transient rTMS after-effects. This method of quantification is widely used as a standard in *in vivo* MRS (Ciurleo et al., 2015). A pre-rTMS screenshot image containing the region of interest (ROI) was used to guide the placement of the post-rTMS screenshot image by a trained MRI Technologist.

### Data Analysis

#### Behavioral and MRS

Task accuracy was calculated by dividing the sum of the number of true positives and negatives by the total number of trials for each subject. Preliminary analysis of variances (ANOVAs) were performed using pre to post RTs, accuracy and metabolite ratios as dependent variables (DVs) with between factor “group” (sham-real, real-sham) and within factor “condition” (sham, real). Due to a non-significant group interaction, “group” was dropped as a factor. Subsequent analyses used paired *t*-tests comparing “pre” and “post” stimulation conditions (Δ_1_; *see equation* 1) as well as *t*-tests comparing sham and real stimulation condition pre to post changes (Δ_2_; *see equation* 2). Δ_1_ was used to determine if changes from baseline were significant for real and sham stimulation separately, while Δ_2_ was used to determine if the changes from baseline for real stimulation differed from sham stimulation.

(1) $$\Delta_{1}=post-pre$$

(2) $$\Delta_{2}=[post_{real}-pre_{real}]-[post_{sham}-pre_{sham}]$$

The reader should note that we chose to use post-pre differences for our DVs to avoid using ANOVA models with invalid main effects. For example, if “time” (pre, post) was a within-subject factor in the same model as “condition” (sham, real)—which was the primary independent variable of interest, the main effect of “condition” would be invalid. This is because half of the data in each mean used in the sham vs. real comparison occurs before we applied stimulation, where no stimulation effect is yet possible. The “condition × time” interaction test of the aforementioned model is identical to the main effect test we performed with “condition” in a model using the pre to post change as the DV. Additionally, the “condition” × “time” interaction test of the aforementioned model is identical to the two-tailed *t*-test we performed for Δ_2_, which is simpler. To test whether baseline levels were the same across all conditions, we performed paired *t*-tests comparing all DVs before real and sham stimulation. Pearson’s correlations were also performed between absolute metabolite ratios as well as between RT and metabolite ratio pre to post changes.

#### fMRI

##### Group analysis

The FMRIB Software Library (FSL v. 4.1.9) was used to initially process the fMRI data used for this study (Smith et al., 2004; Woolrich et al., 2009; Jenkinson et al., 2012). Individual image processing (i.e., first level analysis) was performed in the same manner as described in Parker et al. (2014) for activation and deactivation contrasts. This experimental setup was designed to isolate brain regions used during the encoding phase from the response phase of the SWMT. A second level repeated measures (fixed effects) analysis was used to take the differences between the contrast of parameter estimate (COPEs) images resulting from the initial analyses. The COPEs resulting from the second level analysis were then used as inputs into a third level paired *t*-test (mixed effects) to generate (Real_post_ − Real_pre_) > (Sham_post_ − Sham_pre_) and (Real_post_ − Real_pre_) < (Sham_post_ − Sham_pre_) contrasts. In a separate *post hoc* analysis, paired *t*-tests comparing activation maps before and after stimulation were carried out to generate the following contrasts: Real_post_ > Real_pre_, Real_post_ < Real_pre_, Sham_post_ > Sham_pre_ and Sham_post_ < Sham_pre_ (mixed effects). All above mixed effects analyses used FSL’s “flame 1 + 2” option with de-weighted outliers and were registered to a MNI-152 standard 2 mm atlas.

##### PFC analysis

The PickAtlas software toolbox (Maldjian et al., 2003, 2004) was used to generate a mask for the left DLPFC based on structures from the Talairach Daemon (TD; Lancaster et al., 1997, 2000) and Automated Anatomical Labeling (AAL) databases (Tzourio-Mazoyer et al., 2002). The mask was created by combining the following regions: the inferior and middle frontal gyri TD structures (John et al., 2006; Murray and Ranganath, 2007) and the pars opercularis and pars triangularis of the inferior frontal gyrus AAL structures (Eippert et al., 2007; Krueger et al., 2009, 2011; Telzer et al., 2011). To finalize the mask, these structures were limited to those with MNI coordinates inferior to 40 mm and superior to 12 mm. Thresholded voxel z-statistic values, which were registered to a MNI-152 standard, were extracted using MATLAB (v. 2013a; Mathworks Inc., Natick, MA, USA) and analyzed using SAS (v. 9.2, Cary, NC, USA). A Z and cluster-P threshold of 2.3 and 0.05 were used respectively to identify active voxel clusters (i.e., voxel extent). The pre to post stimulation voxel extent change was correlated with RT as well as each metabolite ratio pre to post stimulation change for the sham and real stimulation conditions separately.

##### Baseline activation maxima

We created a separate spherical mask to determine the location of the most activated voxel that corresponded to the approximate MRS ROI and rTMS site in MNI space (Table 1). The MNI coordinates of the approximate stimulation site were extracted and used as the center in the creation of a spherical ROI with a radius of 20 mm. Each subjects’ baseline imaging scan (transformed into MNI space) was masked with this spherical ROI and the activation maxima (and corresponding MNI coordinates) were extracted.

**Table 1:** Baseline activation maxima within magnetic resonance spectroscopy (MRS) region of interest (ROI)/Approximated rTMS stimulation site.

Subject	Co-ordinates (mm)			Z-max
	*X*	*Y*	*Z*
1	−58	4	28	11.3
2	−50	0	42	10.2
3	−42	6	18	5.8
4	−54	2	40	10.2
5	−42	4	30	7.0
6	−46	18	12	9.0
7	−40	0	24	9.4
8	−36	4	28	10.1
9	−54	−4	38	8.4
10	−40	40	24	9.1
11	−46	12	26	4.5

*Note: these values correspond to the baseline activation maxima for each subject approximately within the ROI used for MRS and rTMS*.

## Results

### Baseline Effects

We did not find significant differences between real and sham conditions for all baseline behavioral and MRS DV measures (*p* > 0.347). This result suggests as whole that biases in working memory performance and neuromolecular levels did not exist in subject populations prior to stimulation, which could potentially influence LF-rTMS after-effects.

### Behavioral

**Figure 2:**
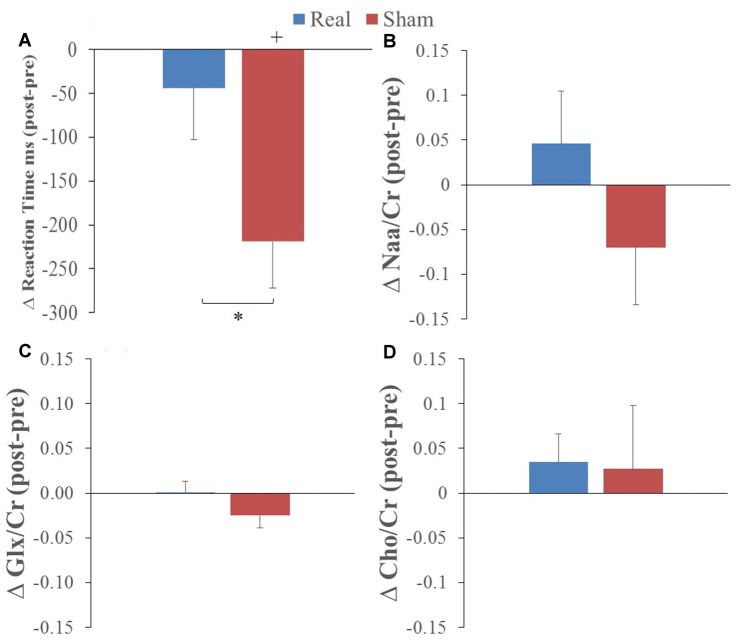
Average pre to post **(A)** reaction time (RT), **(B)** N-acetylaspartate/creatine (Naa/Cr), **(C)** glutamate/glutamine (GlxGlx)/Cr and **(D)** Choline (Cho)/Cr changes following real (blue) and sham (red) stimulation (*corresponds to real vs. sham comparison, paired *t*-test *p* < 0.01; ^+^corresponds to comparison to 0 or no change; one-sample *t*-test *p* < 0.01).

Overall, participants were correct for 90% and 95% of trials when the letter was and was not present, respectively. RT showed a statistically significant decrease from pre to post L-DLPFC LF-rTMS for the sham group only (*t* = −4.12, *P* = 0.002). This pre-post stimulation change was statistically greater for the sham group compared to the real group (*t* = 3.30, *P* = 0.008; see Figure 2A). These results indicate a RT learning effect with task practice and that left DLPFC LF-rTMS abolished this learning effect.

### MRS Main Effects

The only change in metabolites that showed a significant interaction was Cho/Cr (*P* = 0.045) with no main effects (preliminary two-way ANOVA; see “Data Analysis” section). *T*-tests comparing values before and after real and sham stimulation, as well as pre-post differences, did not show any statistical differences for the metabolite ratios (see Figures 2B–D).

### MRS Correlations

**Figure 3:**
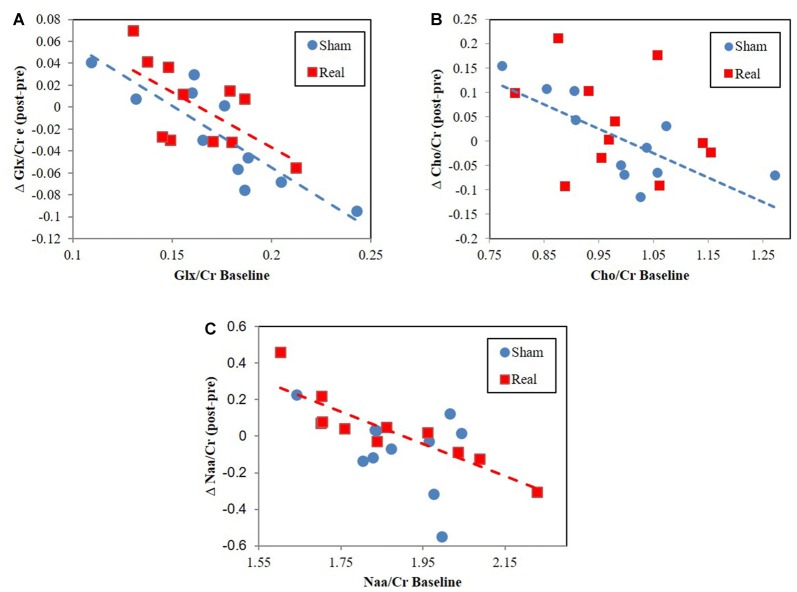
Change from baseline correlation vs. baseline metabolite ratio levels for **(A)** Glx/Cr, **(B)** total NAA (tNAA)/Cr, **(C)** Cho/Cr.

Cho/Cr correlated with tNAA/Cr for both the sham (*r* = 0.78, *P* = 0.004) and real (*r* = 0.68, *P* = 0.022) groups. However, tNAA/Cr significantly correlated with Glx/Cr (*r* = 0.67, *P* = 0.025) for the sham group only. Alternatively, Glx/Cr significantly correlated with Cho/Cr (*r* = 0.71, *P* = 0.014) for the real group only. The change in Glx/Cr from baseline negatively correlated with baseline values for both real (*r* = −0.64, *P* = 0.032) and sham (*r* = −0.87, *P* = 0.0006) conditions (Figure 3A). The change in tNAA/Cr and Cho/Cr from baseline negatively correlated with baseline values for only the real (*r* = −0.89, *P* = 0.0002) and sham (*r* = −0.75, *P* = 0.008) groups respectively (Figures 3B,C). We did not find any correlations between neurometabolites and RT performance.

### fMRI

#### (Post Real − Pre Real) > (Post Sham − Pre Sham) Analysis

The pre to post change in activation following real stimulation was statistically greater than the pre to post change following sham stimulation with centers of gravity (COGs) within the right superior parietal lobule and M1 (cluster 1), left inferior parietal lobule and lateral occipital cortex (LOC; cluster 2) as well as the paracingulate gyrus (cluster 3; Table 2; Figure 3). Cluster 1 included additional brain regions with local maxima within the left superior parietal lobule and right somatosensory cortex (SI; BA3a).

**Table 2:** Group activations during Sternberg task following repetitive transcranial magnetic stimulation (rTMS).

Cluster	Brain region	*P*	Z-max	Cluster COG (mm)			# Voxels
				*X*	*Y*	*Z*
	*(Post Real − Pre Real) > (Post Sham − Pre Sham)*						
1	R, primary motor cortex (BA4a)	0.000	3.8	2.2	−43.3	61.8	855
2	L, lateral occipital cortex	0.001	3.6	−38.9	−70.8	25.8	376
3	L, paracingulate gyrus	0.003	3.7	3.5	52.5	5.0	330
	*Post Sham < Pre Sham*						
3	L, paracingulate gyrus	0.018	3.5	4.0	50.0	10.0	257
	*Post Real > Pre Real*						
1	R, primary motor cortex (BA4a)	0.013	3.4	0.2	−46.6	58.2	352
4	L, frontal pole	0.027	3.5	−25.6	50.5	28.3	311
2	L, lateral occipital cortex	0.029	3.4	−19.3	−76.4	42.7	307

*Note: only significant clusters (*P* < 0.05; paired t-test) are displayed for indicated contrasts. (Post Real − Pre Real) > (Post Sham − Pre Sham) contrast values correspond to brain regions with a larger activation change from baseline following real stimulation compared to sham. Post Sham < Pre Sham and Post Real > Pre Real contrast values correspond to decreases and increases in activation values within brain regions following sham and real stimulation respectively. Center of gravity (COG) coordinates are given in MNI space*.

#### Pre- vs. Post-rTMS Analysis

**Figure 4:**
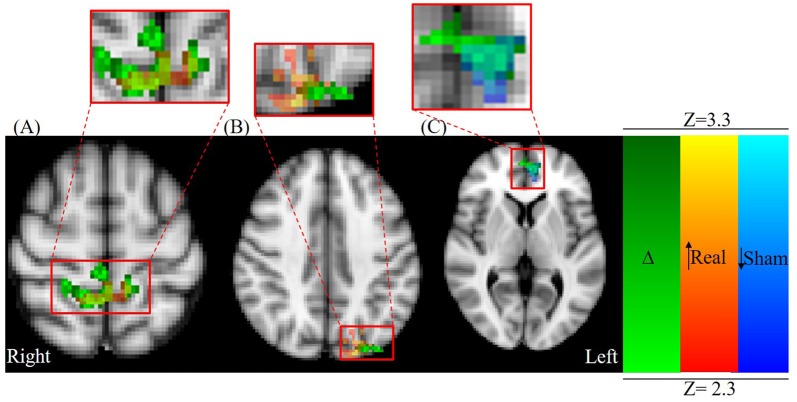
Axial view centered on overlapping contrast images: green; (Δ) = (*Post Real − Pre Real) > (Post Sham − Pre Sham)* red-yellow; (Real) = *Post Real > Pre Real*, and blue-light blue; (Sham) = *Post Sham < Pre Sham*. **(A)** Parietal lobule/primary motor cortex (M1) activation (MNI: *X* = 2.16 mm, *Y* = −43.3 mm, *Z* = 61.8 mm) and **(B)** occipital cortex activation changes (MNI: *X* = −40.0 mm, *Y* = −80.0 mm, *Z* = 40.0 mm) are primarily a result of significant activation changes following real stimulation (red-yellow colormap). **(C)** Paracingulate gyrus/ACC changes (MNI: *X* = −3.48, *Y* = 52.5, *Z* = 4.95 mm) are primarily a result of significant activation changes following sham stimulation (blue-light blue colormap).

*Post hoc* paired *t*-tests indicated that clusters 1 and 2 resulted from real stimulation while cluster 3 occurred in the sham stimulation condition (Table 2; Figure 4). Statistically significant clusters 1 and 2 from the above third-level analysis therefore likely resulted from larger pre to post increases in activation (or decreases in deactivation) when comparing real to sham stimulation. Conversely, third-level cluster 3 likely resulted from smaller pre to post decreases in activation (or increases in deactivation) when comparing real to sham stimulation conditions. In addition to paracingulate gyrus activation changes, local maxima within the anterior cingulated cortex were found in the sham stimulation condition. *Post hoc* analysis identified an additional cluster, cluster 4 (Table 2; not shown), which lies within the left DLPFC following real stimulation.

## Discussion

We investigated neuromolecular and activation changes following a single session of neuronavigated 1 Hz left DLPFC rTMS in healthy participants. The main findings can be summarized as follows: (1) single session LF-rTMS was not sufficient to cause changes in absolute neurometabolite ratios in the left DLPFC. (2) Absolute neurometabolite ratio changes did not correlate with activation changes. (3) Single session LF-rTMS was sufficient to induce changes in metabolite ratio correlations in the left DLPFC. (4) LF- rTMS abolished SWMT learning effects. Lastly, (5) rTMS-abolished learning effects were associated with increased engagement of alternate remote brain regions.

### Single Session Not Sufficient to Cause Changes in Absolute Metabolite Ratio Changes

The lack of statistical significance when comparing pre and post neurometabolite ratios following real stimulation suggests that single session LF-rTMS is not sufficient to cause measurable changes using our imaging paradigm as originally hypothesized. This also includes comparisons of neurometabolite ratio changes and fMRI activation changes. A majority of rTMS human MRS studies (Luborzewski et al., 2007; Fregni et al., 2011; Yang et al., 2014) and low frequency molecular-based rTMS rodent studies (Liebetanz et al., 2003; Aydin-Abidin et al., 2008; Trippe et al., 2009; Yue et al., 2009; Tan et al., 2013) showed molecular changes occurred after multiple sessions of stimulation. Quantifiable neurometabolite changes using current methods may therefore require multiple stimulation sessions (as opposed to a single session). Michael et al. (2003), however, did find significant stimulation site Glx concentration changes following a single session of rTMS. While their study used a stimulation site similar to the one used in this study, they used a significantly higher stimulation frequency (20 Hz), which may have stronger or more rapidly occurring after-effects. Further, the acute mechanisms of high frequency stimulation are likely different than that of low frequency stimulation (Valiulis et al., 2012). Given that a single stimulation session was not sufficient to cause measurable MRS and activation measure changes, it is not surprising that neurometabolite ratio changes did not correlate with activation changes as originally hypothesized. This lack of correlation may also be due to the fact that fMRI measures were collected after MRS imaging. More specifically, Glx and tNAA-related effects may have no longer been present when imaging took place. These transient effects may have been followed by changes in other neurotransmitters not captured using MRS, such as changes in dopamine, which has been shown in humans following HF-rTMS (Cho and Strafella, 2009). Future studies, should therefore employ simultaneous imaging modalities such as fMRI-Positron Emission Tomography (Wey et al., 2014) or fMRI-MRS (Betina Ip et al., 2017).

### Single Session Sufficient to Induce Changes in Metabolite Ratio Correlations

While we did not find a global change in metabolite ratios across participants, we did find that pre to post metabolite ratios negatively correlated with baseline metabolite ratio levels (Figure 3). This result, also found for Glx following HF DLPFC stimulation (Michael et al., 2003), indicates that changes occurred following real and sham stimulation but that these changes depended on baseline levels. Interestingly, the magnitude and directionality of metabolite ratio changed depending on where baseline levels were relative to a central concentration point/region (Figure 3). The lower the values were relative to this central point, the more likely it was for values to increase as a result of the stimulation. The opposite was true for higher baseline values. Cho/Cr and Glx/Cr changes may be a type of homeostatic-like response to rTMS as study participants engage working memory centers. These negative Cho correlations were not present during real rTMS, which suggests that rTMS interferes with pathways driving Cho level changes. Conversely, tNAA negative correlation changes only existed following real stimulation. rTMS in this case may engage alternate pathway(s) that result in tNaa/Cr level baseline relationships.

Research shows that NaaG and Glu are synthesized from NAA (Becker et al., 2010; Long et al., 2013). Given this relationship and that tNAA (comprised of NAA and NAAG) and Glx correlate during sham but not real stimulation, it is possible that rTMS interferes with the processes associated with NAAG synthesis. A higher magnetic field strength and specialized analyses, however, are necessary to parse out NAA and NAAG level changes that comprise the tNAA peak. Given that Cho is a marker of cell turnover, our results suggest that LF-rTMS influences cell membrane synthesis. Reasons behind the observed Cho/Cr relationships, however, are unclear and warrant additional investigation. In general, our findings provide evidence that rTMS changes the way tNAA, Cho and Glx relate. More work should be devoted to investigating these relationships in future studies.

It is likely that regression towards the mean (RTM) effects are contributing to the correlations we found between baseline neurometabolite ratios and rTMS-induced changes of those ratios. If this was the only effect, however, we would expect significant correlations to exist in both the real and sham stimulation conditions as this effect would apply equally across stimulation conditions. This is especially the case since we did not find significant differences between real and sham conditions for all baseline DV measures (*p* > 0.347). In our case, however, correlation relationships differed for real and sham conditions.

### Task Learning Effect Abolished Following Real Stimulation

We observed a learning effect associated with the SWMT, which was illustrated by a significant improvement in RT performance following sham stimulation. Additionally, fMRI analyses revealed that learning following sham stimulation was accompanied by decreased activity in the paracingulate gyrus (i.e., cluster 3; Table 2), which is a part of the dorsal portion of the anterior cingulate cortex (ACC; Metzak et al., 2012). This aligns with previous research showing that decreased ACC activity is associated with decreased effort (Bunge et al., 2001; Jansma et al., 2007) and practice (Jansma et al., 2007) in the SWMT (van Raalten et al., 2008). As hypothesized, LF-rTMS was sufficient to abolish this learning effect which is reflected by a nonsignificant change in RT performance in the real stimulation condition only. This is consistent with research showing the LF-rTMS inhibits working memory performance in some cases (Škrdlantová et al., 2005; Weigand et al., 2013), but contrasts others where LF-rTMS either increased performance (Fregni et al., 2006a; Fitzgerald et al., 2009) or had no effect (Hoffman et al., 2005; Kang et al., 2009; Kim et al., 2010; Watts et al., 2012). Interestingly, all studies showing decreased performance—to include ours, were conducted in healthy humans, wheareas studies not showing this decreased performance were conducted in subjects with neurological disorders/psychiatric illnesses. Specifically these studies investigated rTMS-effects in patients with depression (Fitzgerald et al., 2009), epilepsy (Fregni et al., 2006b), obsessive-compulsive disorder (Kang et al., 2009), stroke (Kim et al., 2010), and post-traumatic stress disorder (Watts et al., 2012), which have all been associated with abnormal neurotransmitter levels (Abnormalities and Obsessive, 2012; Sanacora et al., 2012; Xing et al., 2012; Yang, 2014; Barker-Haliski and White, 2015; Häge et al., 2016). Therefore, future research relating neurotransmitter levels in healthy and non-healthy populations following DLPFC LF-rTMS may help shed light on the varying behavioral effects seen across literature.

### Real Stimulation Resulted in Increased Engagement in Target and Remote Brain Regions

Increased activation at the target DLPFC contrasts our hypothesis. We expected decreased BOLD activation to parallel decreased oxygenated hemoglobin levels following prefrontal LF-rTMS (Kozel et al., 2009), as BOLD and oxygenated hemoglobin measures are highly related (Scarapicchia et al., 2017). Kozel et al. (2009), however, did not employ a task that engaged working memory centers as is the case in our study. Given the increasing evidence that neuromodulation-effects are state dependent (Silvanto, 2008; Silvanto and Cattaneo, 2014) it is possible that engaging the DLPFC during SWMT interacted with rTMS after-effects such that we saw opposite activation effects as expected.

In terms of networks specifically engaged in the SWMT (van Raalten et al., 2008) identified function-specific changes across the cortex that relate to SWMT-practice in the encoding phase using fMRI (van Raalten et al., 2008). Investigators found that the bilateral occipital and superior parietal cortices as well as the dorsal portion of the ACC, left DLPFC and putamen were activated by the task. Each of these regions showed decreased activity with SWMT-practice as RT performance improved over time. Given that we applied inhibitory rTMS to the left DLPFC, it is possible that the stimulation may have disrupted the natural learning processes associated in engaging these brain regions. In support of this idea, while we did find changes in dorsal ACC activity in the sham stimulation as in van Raalten et al. (2008), we did not see such changes following real rTMS. Additionally, rather than seeing decreases in occipital and DLPFC activity as in van Raalten et al. (2008), we saw increased activation in these regions as well as in the M1.

In fact, increased motor, occipital and DLPFC activation following DLPFC stimulation may reflect a redistribution of cognitive resources to the motor, somatosensory and visual cortices. This idea falls in line with a widely accepted working memory model described in Liao et al. (2014) and developed by Baddeley and Hitch (1974). The model proposes that executive brain regions exert supervisory control over attentional resource allocation within the “the phonological loop that manages verbalizable content” and a “visuo-spatial sketchpad that manages visual content”. Increased M1 activity, in this case, may result from participants engaging verbal memory rehearsal mechanisms. Research showing that M1 rTMS influences RT SWMT performance supports this idea (Liao et al., 2014). Similarly, increased LOC activation, which is known to involve object recognition (Grill-Spector et al., 2001) and present a linear load dependence in SWMTs (Metzak et al., 2012), may result from increased use of visual processing resources. DLPFC-visual cortex related increases is further supported by a study showing participants who increased DLPFC activity using feedback from real-time fMRI also showed middle occipital cortex activity in a 3-back working memory task (Zhang et al., 2013).

### Limitations

It is important to note the time course of imaging in this study. Studies finding decreased BOLD effects following DLPFC stimulation typically perform fMRI immediately after stimulation (Jansma et al., 2013; van den Heuvel et al., 2013; Gerrits et al., 2015). As our primary focus was on quantifying neurometabolites, fMRI was performed after MRS acquisition (i.e., >20 min following stimulation). Given, the relatively low intensity and short duration of stimulation we may have seen a detectable decrease in activity if fMRI was performed closer to the end of stimulation. Additionally, the 1.5 T magnetic field strength makes it difficult to parse out components of the Glx signal, which may also comprise spectra for the inhibitory neurotransmitter GABA (Maddock and Buonocore, 2012). GABA, however, typically has significantly lower concentrations (Rowland et al., 2013) and contributes a small portion to the Glx signal (Maddock and Buonocore, 2012). In regards to Cr normalization, it is possible that the observed correlational changes were due to altered Cr levels, which could be affected by rTMS-altered metabolism rather than changes in tNAA, Glx and Cho levels themselves. If Cr levels primarily drove the effects we observed, however, we would expect changes to be same across the normalized metabolites, which was not the case. Additionally, while rTMS-induced Cr changes are possible, the magnitude of change is likely small as Cr levels are stable over short term repeated measurements (Buonocore and Maddock, 2015). This suggests that while Cr itself may have changed somewhat following rTMS, the effects were moreso driven by tNAA, Glx and Cho levels. Finally, we acknowledge that a larger sample size would be ideal—which should be taken into consideration when interpreting results. However, the results reported herein provide important initial evidence of rTMS effects on metabolites that should be an area for further exploration in future experiments.

## Conclusion

In conclusion, our results show that a single session of DLPFC LF-rTMS is sufficient to influence the relationship between measures of tNAA, Glx and Cho. This is both in comparison to baseline values, which are negatively correlated and between the measures themselves. Changes in tNAA:Glx and Cho relationships suggest that LF-rTMS influences NAAG synthesis and cell turnover respectively. Imaging using higher magnetic field strengths and data processing optimized to investigate these relationships should be considered in future studies. Our results also show that a single session was capable of abolishing SWMT-related learning effects >20 min from stimulation and that these effects were associated with an increased engagement of the M1, SI, LOC. More research, however, is needed in investigating acute effects of LF-rTMS in healthy humans. Such knowledge would improve the efficacy of LF-rTMS in clinical populations as well as expand understanding in research areas investigating cognition in healthy humans. Importantly, this study highlights that investigators should not only research absolute neuromolecular changes but consider how rTMS changes relationships between these molecules.

## Author Contributions

RAM and JTN conceived the study. NRB, RAM, JGP, CF, NA, AM and JTN contributed to the design of the study. NRB, AM, DB, JGP, LKM, JTN, CF, NA and AM contributed in some form or another to either applying TMS and/or procedures related to the MRI acquisition process. NRB, RAM, DB, MSS and CG performed the analysis and/or interpretation of results presented in this work. NRB, RAM, DB, MSS, JGP, LKM, JMN, CF, NA, AM, CG and JTN contributed to either the drafting and/or document revision as well as provided final approval and agree to be accountable to all aspects of the work.

## Distrubution Statement A

Approved for public release: distribution is unlimited. 88ABW Cleared 02/22/18; 88ABW-2018-0824.

## Conflict of Interest Statement

NRB, LKM, JMN and CG were employed by INFOSCITEX, a dcs company. The other authors declare that the research was conducted in the absence of any commercial or financial relationships that could be construed as a potential conflict of interest.

## Abbreviations

COPE: contrast of parameter estimate
DLPFC: dorsolateral prefrontal cortex
LF-rTMS: low frequency repetitive transcranial magnetic stimulation
SWMT: sternberg working memory task
WM: working memory.
